# Co_2_FeGe Heusler Alloy Nanoparticle Catalysts for Propyne Hydrogenation and Ammonia Decomposition

**DOI:** 10.1002/open.202300131

**Published:** 2023-11-06

**Authors:** Takayuki Kojima, Yuki Nakaya, Souta Tate, Satoshi Kameoka, Shinya Furukawa

**Affiliations:** ^1^ Division of Chemistry and Materials Faculty of Textile Science and Technology Shinshu University 3-15-1, Tokida Ueda Nagano 386-8567 Japan; ^2^ Institute for Catalysis Hokkaido University N21, W10 Sapporo 001-0021 Japan; ^3^ Institute of Multidisciplinary Research for Advanced Materials Tohoku University 2-1-1, Katahira, Aoba-ku Sendai 980-8577 Japan; ^4^ Present address: Division of Applied Chemistry Graduate School of Engineering Osaka University (Japan)

**Keywords:** intermetallic catalyst, intermetallic compound, Heusler alloy, Heusler compound

## Abstract

Heusler alloys (X_2_YZ) can be a candidate for new catalysts as well as other intermetallic compounds. We previously found good catalytic properties of Co_2_FeGe for selective hydrogenation of alkynes and developed nanoparticles of Co_2_FeGe supported on SiO_2_. However, the average diameter of the nanoparticles was 23 nm, which is not small enough compared to those of state‐of‐the‐art nanoparticle catalysts. In this study, we developed SiO_2_‐supported Co_2_FeGe nanoparticles of <10 nm in diameter. A catalytic test for selective hydrogenation of propyne indicated a partial formation of sites with low selectivity including excess Co atoms. For ammonia decomposition, enhancement of turnover frequency was achieved by reducing the particle size.

## Graphical abstract

Co_2_FeGe Heusler alloy nanoparticle catalysts of <10 nm in diameter were synthesized. Reaction rate per weight (circles) and turnover frequency (TOF, triangles) for NH_3_ decomposition (red) were monotonically increased by reducing particle size from 23 nm to 7.5 nm, while those and C_3_H_6_ selectivity (cross) for C_3_H_4_ hydrogenation (green) did not follow the same monotonic trend.

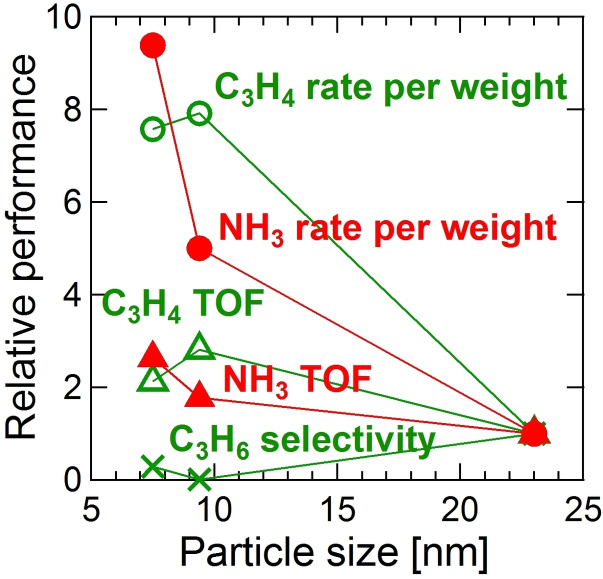

## Introduction

Intermetallic compounds exhibit unique catalytic properties because of their unique electronic structures and surfaces of chemically ordered crystal structures.[^1^, ^2^, ^3^, ^4^, ^5^] Ternary intermetallics with the L2_1_‐type structure, called “Heusler alloys” (or “Heusler compounds”), can be new catalysts, because various sets of X, Y, and Z can be chosen and properties can be controlled by partial elemental substitution.^[6]^ Their catalytic properties have been investigated for various reactions such as hydrogenation of alkynes,[^6^, ^7^] oxidation of carbon monoxide,^[7]^ steam reforming of methanol,^[8]^ hydrogenation of carbon dioxide,[^9^, ^10^] dehydrogenation of 2‐propanol,^[11]^ electrochemical oxygen evolution reaction,^[12]^ and electrochemical reduction of carbon dioxide.[^13^, ^14^] For example, Co_2_FeGe showed excellent alkene selectivity for selective hydrogenation of alkynes.^[6]^ In this previous study, metallurgically synthesized powders with particle sizes on the order of microns were used as catalyst samples due to ease of synthesis and high reproducibility.^[15]^ Thus, Co_2_FeGe was then downsized by developing SiO_2_‐supported catalysts.^[16]^ For selective hydrogenation of propyne (C_3_H_4_), SiO_2_‐supported Co_2_FeGe showed a 2,000 times larger reaction rate per weight of Co compared to micron powders while high propene (C_3_H_6_) selectivity (>70 %) was kept. Its average particle size was estimated to be 23 nm, however, which could be downsized even more to less than 10 nm as other intermetallic catalysts.[^17^, ^18^] In this report, we synthesized smaller Co_2_FeGe nanoparticles supported on SiO_2_ than the previous one. In addition to the C_3_H_4_ hydrogenation, ammonia (NH_3_) decomposition was used as a model reaction for investigating catalytic properties of the nanoparticles, because it is a structure‐sensitive reaction in that the turnover frequency (TOF) depends on the particle size.^[19]^

## Experimental Section

SiO_2_‐supported Co_2_FeGe catalysts were prepared by a pore‐filling impregnation method.^[16]^ Co(NO_3_)_3_ ⋅ 6H_2_O (Wako, 98 %), Fe(NO_3_)_3_ ⋅ 9H_2_O (Sigma‐Aldrich, 98 %), (NH_4_)_2_GeF_6_ (Aldrich, 99.9 %) were dissolved in deionized water, in which a molar ratio was Co : Fe : Ge=1.8 : 1 : 1. The precursor solution was dropped to ground dried silica‐gel (CARiACT G‐6, Fuji Silysia), in which the amount of solution was equal to a pore volume of the silica‐gel. The mixture was kept in a sealed round‐bottom flask overnight at room temperature. It was quickly frozen using liquid nitrogen, followed by vacuum freeze‐drying at 0 °C. After further drying it in an oven overnight at 90 °C, the resulting powder was calcined in dry air for 1 h and finally reduced by H_2_ at 800 °C for 1 h. Three samples were synthesized by different amounts of Co loaded (*w*_Co_) and calcination temperature (*T*_cal_): sample 1) *w*_Co_=3 wt%, *T*_cal_=500 °C; sample 2) *w*_Co_=1 wt%, *T*_cal_=400 °C; sample 3) *w*_Co_=0.5 wt%, *T*_cal_=400 °C. Sample 1) has been already characterized to be Co_45.2_Fe_29.4_Ge_25.4_ with L2_1_‐structural ordering of 80 % in the previous report.^[16]^

The crystallite structure was analyzed by synchrotron powder X‐ray diffraction (XRD) in transmission geometry using a glass capillary sample holder of 0.3 mm diameter. An incident X‐ray energy was 25 keV. The crystallite size was estimated by the Scherrer equation as same as in the previous report.^[16]^ Sample morphology was investigated by transmission electron microscopy (TEM, JEOL JEM‐2010). Information about surface area and active sites was obtained by pulse adsorption of CO gas (2 % CO/98 % He). After saturation of adsorption, temperature‐programmed desorption of CO (CO‐TPD) at a heating rate of 10 °C min^−1^ was performed using mass spectroscopy (Pfeiffer Prisma QMS 200 M1).^[11]^

Catalytic properties were evaluated in a standard flow reactor. The C_3_H_4_ hydrogenation was conducted as same as in the previous report.^[16]^ After heating under H_2_ gas flow at 800 °C for 1 h, a gaseous mixture of [0.1 % C_3_H_4_/40 % H_2_/59.9 % He] was introduced at a flow rate of 30 mL min^−1^. From room temperature to 250 °C, the products were analyzed by gas chromatography (Agilent 490 Micro GC with PoraPLOT Q column) after waiting 30 min at each temperature. NH_3_ decomposition was also performed. After heating under H_2_ gas flow at 800 °C for 1 h, the reactor was flushed by He gas (50 mL min^−1^) for 10 min at 300 °C to remove hydrogen species adsorbed on a catalyst surface. The reaction was conducted using a gaseous mixture of [1 % NH_3_/99 % He] at a flow rate of 30 mL min^−1^ from 300 °C to 600 °C. The products were analyzed by gas chromatography (GL Science GC 3210 equipped with a thermal conductivity detector and two packed columns (outer diameter: 1/8 inch, length: 2 m): Porapak N in Sulfinert® tube for NH_3_ and Molecular Sieve 5 A for N_2_ and H_2_) using He carrier gas. A TOF in s^−1^ was calculated to be a reaction rate in mol s^−1^ g(Co)^−1^ divided by the number of CO molecules in mol g(Co)^−1^ in the pulse adsorption.

## Results and Discussion

**Figure 1:**
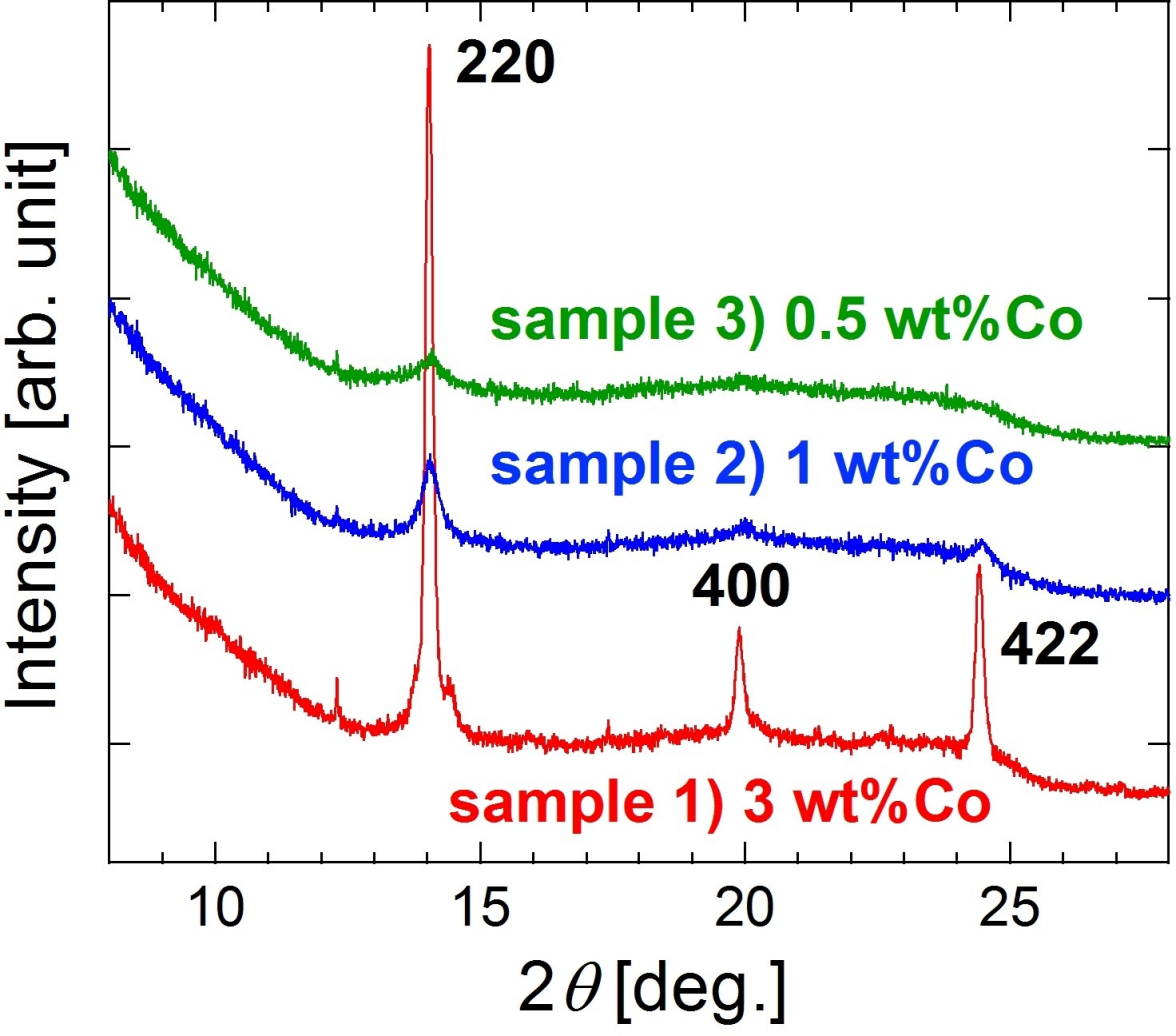
XRD patterns for SiO_2_‐supported Co_2_FeGe nanoparticles, sample 1–3). The peak at around 2*θ*=12° possibly originates from an oxide formed in air.

Figure 1 shows XRD patterns. In principle, superlattice peaks are too weak in this material consisting of elements with similar atomic numbers because their peak intensities are proportional to the square of the difference in atomic numbers. In Figure 1, thus, 111 and 200 superlattice peaks at 2*θ*=8.6° and 9.9°, respectively, are not visible, detection of which was made more difficult due to peak broadening by nanosizing. Although a peak possibly of CoGe was detected at the side of the 220 Co_2_FeGe peak for sample 1) as reported previously,^[16]^ almost a single phase of Co_2_FeGe was likely obtained in all samples. The Heusler phase was maintained even when the amount of metals loaded was decreased to reduce the particle size. The crystallite size was estimated from the 220 peak width using the Scherrer equation and is listed in Table 1. It was reduced from 15 nm for sample 1) to 4.9 nm for sample 3). The average particle size of sample 1) was previously estimated to be 23 nm from scanning TEM images.^[16]^

**Table 1:** Characteristics of samples 1–3). Particle size of sample 1) is the average value estimated by scanning TEM in a previous report,^[16]^ while those of samples 2) and 3) are the product of crystallite size and 23/15 (size ratio of particle and crystallite in sample 1). Surface area was calculated from particle size and density on the assumption of spherical shape.

Sample No.	Co loading [wt%]	Crystallite size [nm]	Particle size [nm]	Surface area [m^2^ g^−1^]	CO adsorbed [10^−5^ mol g(Co)^−1^]	CO adsorbed [10^−6^ mol m^−2^]	TOF [s^−1^] (C_3_H_4_, 50 °C)	TOF [s^−1^] (NH_3_, 450 °C)
1	3	15	23	30	8.1	1.2	0.018	0.30
2	1	6.2	9.4	73	23	1.4	0.052	0.53
3	0.5	4.9	7.5	93	29	1.4	0.039	0.79

**Figure 2:**
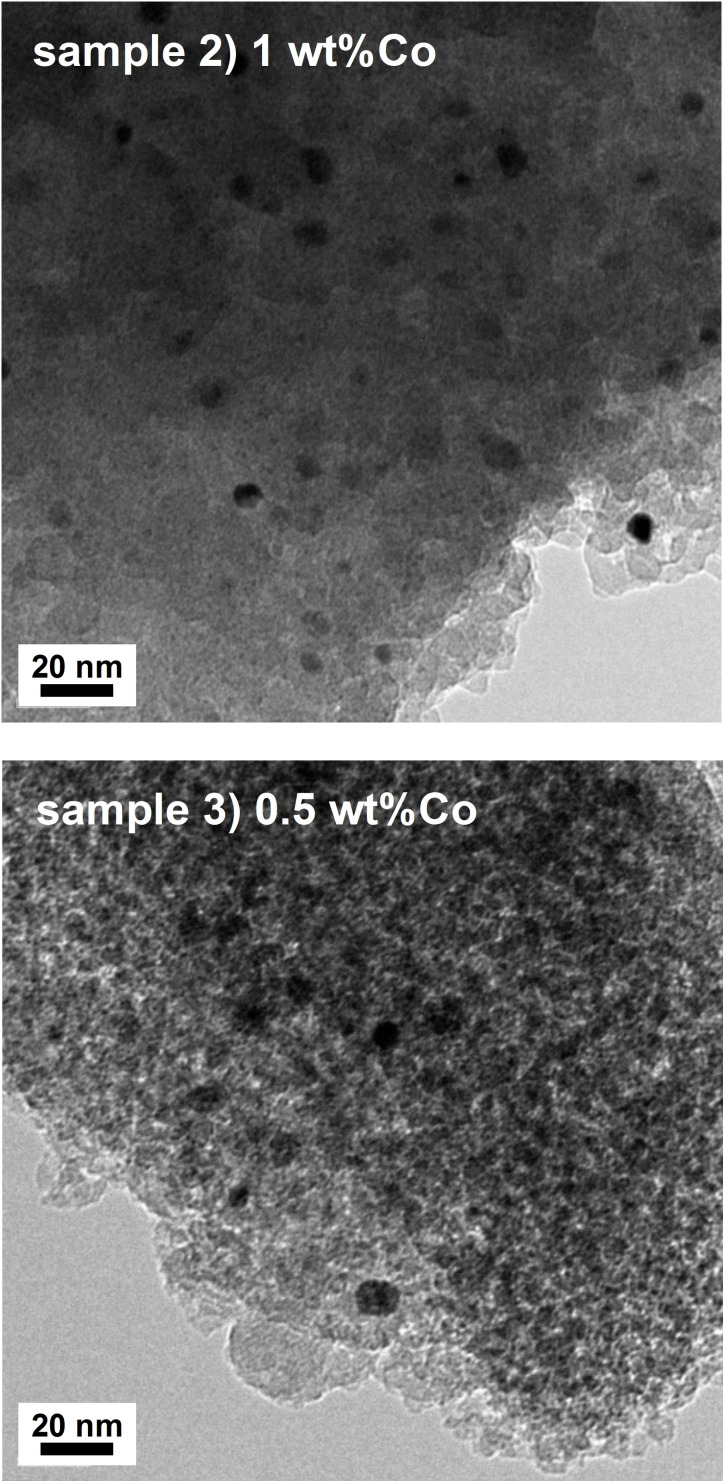
TEM images for samples 2) and 3). Co_2_FeGe particles can be seen as dark particles dispersed on SiO_2_ matrix.

Assuming that the average size ratio of crystallites and particles is 15 : 23 also in other samples, the particle size was estimated to be 9.4 nm and 7.5 nm for samples 2) and 3), respectively. Particles around these sizes were actually observed in TEM images as shown in Figure 2 (A rough estimate of size distribution is provided in the Supporting Information). From these particle sizes and the density (8.66 g cm^−3^),^[16]^ the surface area of Co_2_FeGe particles was roughly estimated on the assumption of spherical particles. The amount of CO molecules adsorbed in the pulse adsorption was divided by the surface area as listed in Table 1. Their values in mol m^−2^ are similar for all samples; thus, the estimated particle sizes of samples 2) and 3) in Table 1 are likely valid.

**Figure 3:**
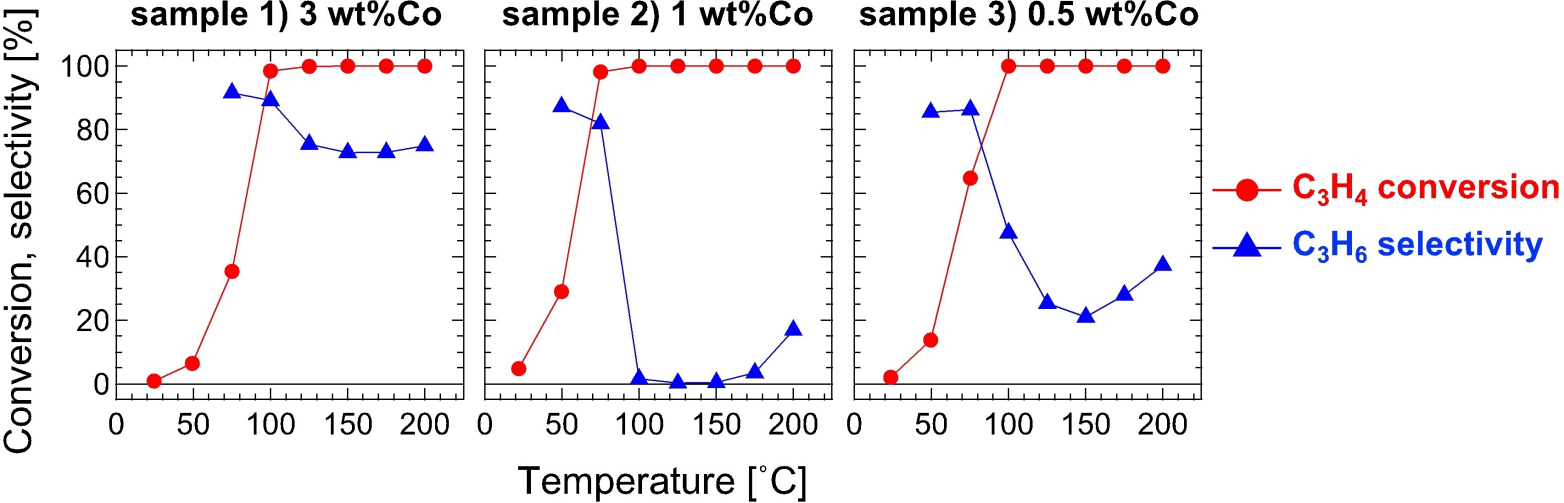
C_3_H_4_ conversion and C_3_H_6_ selectivity in C_3_H_4_ hydrogenation by catalyst samples 1–3). Weights of catalyst used are 30 mg for sample 1) and 50 mg for samples 2) and 3). Carbon loss due to side reactions was negligible in all samples. Data for sample 1) was reproduced from Ref. [16] licensed under CC BY 3.0 (https://creativecommons.org/licenses/by/3.0/).

Figure 3 shows catalytic properties for the C_3_H_4_ hydrogenation. In general, the alkene selectivity tends to be high when the alkyne conversion is less than 100 % because the alkene products desorbed from catalyst surfaces hardly adsorb again due to alkyne molecules adsorbing strongly on the surfaces.[^6^, ^20^, ^21^] All the samples in Figure 3 followed this tendency. After the C_3_H_4_ conversion reached 100 %, sample 1) kept a high C_3_H_6_ selectivity over 70 %; however, samples 2 and 3) lost the selectivity, which indicated the existence of low‐selective sites on the catalyst surfaces.

**Figure 4:**
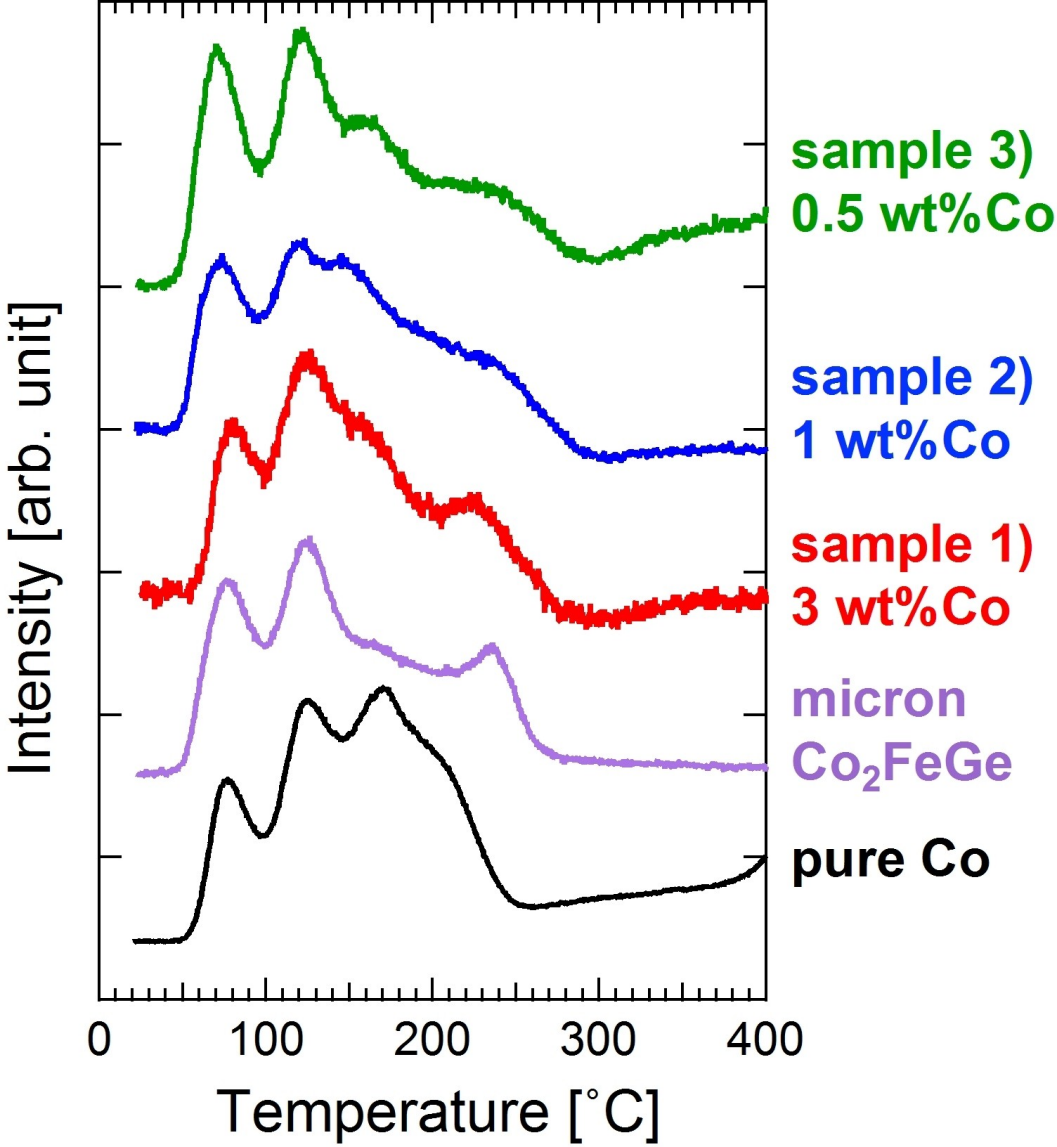
CO‐TPD profiles for samples 1–3), micron powder of Co_2_FeGe (<20 μm), and pure Co powder (1–2 μm). Data for sample 1), micron Co_2_FeGe, and pure Co were reproduced from Ref. [11] with permission from The Royal Society of Chemistry.

Figure 4 shows CO‐TPD profiles. Peaks at 70–80 °C and 120 °C were commonly observed for samples 1–3), the micron powder, and pure Co powder. The pure Co also exhibited a peak at 170 °C, signals corresponding to which were observed for samples 1–3) but not observed for the micron powder. In the previous report,^[16]^ this signal was large for a sample with a molar ratio of Co : Fe : Ge=2 : 1 : 1, C_3_H_6_ selectivity of which was very low compared to the sample 1) with Co : Fe : Ge=1.8 : 1 : 1. This signal that seems to be originated from monometallic Co sites was clearer for the samples 2) and 3) than the sample 1). A peak at 230–240 °C observed for the micron powder was attenuated for samples 2) and 3), which also made the profiles similar to that for the pure Co. Thus, catalytically active sites consisting of excess Co atoms likely formed due to inhomogeneous mixing in synthesis processes or structural disordering. However, the TOF of C_3_H_4_ was not so different among the samples 1–3) as listed in Table 1, which indicates that C_3_H_4_ molecules were hydrogenated to C_3_H_6_ mainly on the intended active sites without excess Co atoms and the C_3_H_6_ molecules were further hydrogenated to C_3_H_8_ on a small amount of the excess Co active sites.

**Figure 5:**
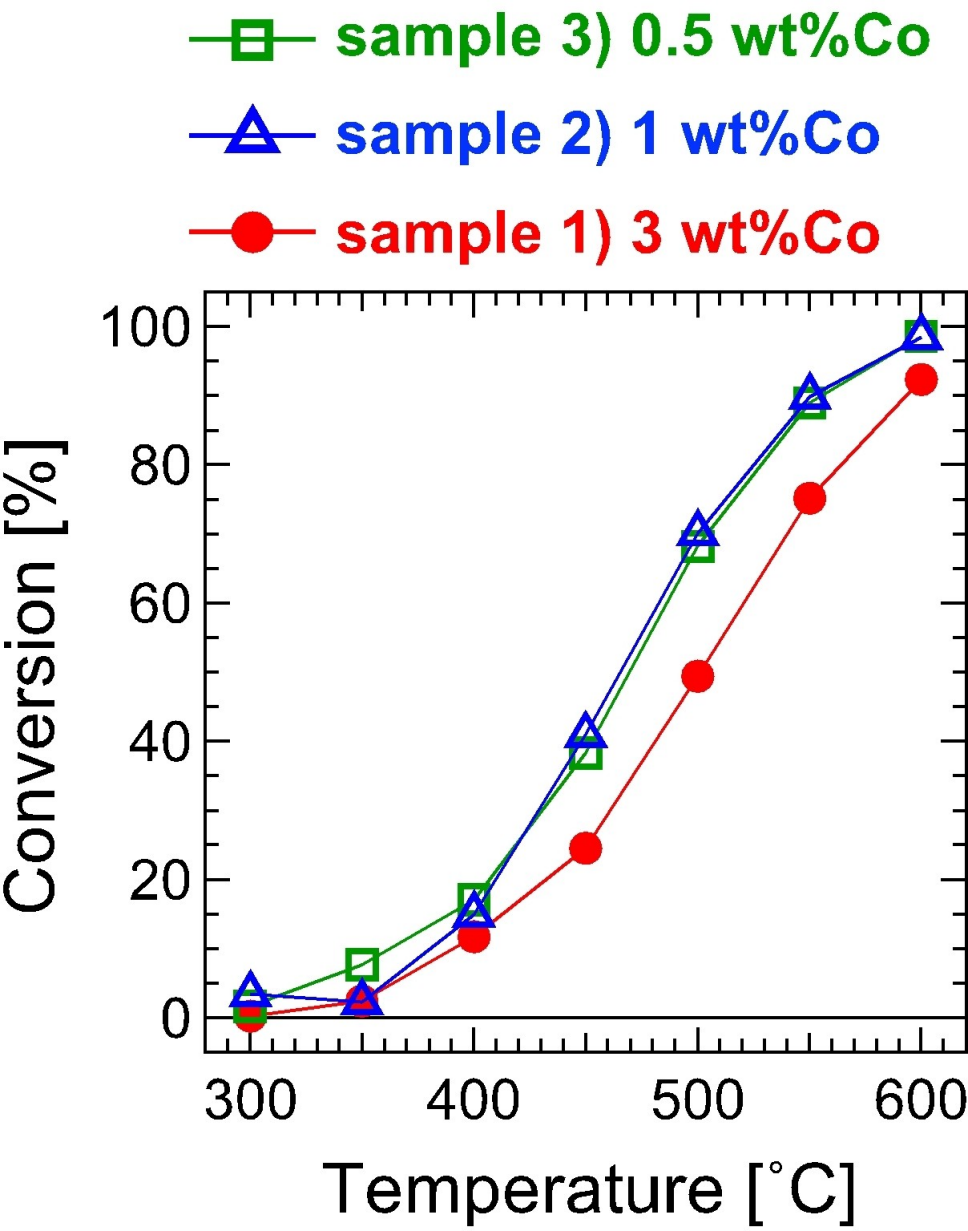
Conversion in NH_3_ decomposition by catalyst samples 1–3). Weights of catalyst used are 70 mg for all samples.

Figure 5 shows the conversion for the NH_3_ decomposition. Samples 2) and 3) showed a higher conversion than sample 3). The conversions by samples 2) and 3) were almost the same even though sample 3) contains half as much metal as sample 2).

Thus, TOF monotonically increased with a reduction in the particle size as shown in Table 1. The structure sensitivity of NH_3_ decomposition has been reported as TOF by Ru catalysts depends on the particle size, in which several nanometers give the maximum TOF.^[19]^ The active site, the so‐called B_5_ site, includes low‐coordinated Ru atoms.^[22]^ The increase in TOF from sample 1) to sample 3) likely originates from an increase in low‐coordinated atoms with the reduction in the particle size.

By reducing the particle size, TOF was increased for the NH_3_ decomposition; however, the C_3_H_6_ selectivity was decreased for the C_3_H_4_ hydrogenation due to the undesirable active sites likely formed by inhomogeneous mixing in synthesis or structural disordering. The ordered structure is destabilized by nanosizing due to an increase in the number ratio of surface atoms to bulk atoms.^[23]^ For example, L1_0_‐type CoPt disorders to A1 (fcc) phase at 825 °C in bulk state, while its particles with a diameter of several nanometers disorders at 500–650 °C.^[24]^ However, Co_2_FeGe does not disorder below the melting point (1128 °C).[^25^, ^26^] The particle size is not too small (7.5 nm in sample 3). Thus, the disordering temperature is not likely to be significantly reduced as much as that the ordered phase cannot be obtained. Nevertheless, the stability in samples 2) and 3) is certainly reduced from that in the bulk and sample 1). Further optimization is needed to synthesize Co_2_FeGe nanoparticles of <10 nm in diameter with negligible disordering and segregation.

## Conclusions

SiO_2_‐supported Co_2_FeGe Heusler alloy nanoparticles with different particle sizes were synthesized by changing the amount of metals loaded in the impregnation process. The XRD analysis, the TEM observation, and the CO pulse adsorption indicated that the average particle size was reduced from 23 nm to 7.5 nm. For the C_3_H_4_ hydrogenation, the TOF was not so different among the three samples, while the C_3_H_6_ selectivity was low in the samples with the particle size of <10 nm. The CO‐TPD indicated that the low selectivity was due to the active sites including excess Co atoms. For the NH_3_ decomposition, the TOF monotonically increased with the reduction in particle size. This increase was considered due to the increase in low‐coordinated atoms according to the literature.[^19^, ^22^] This study basically succeeded in synthesizing the Co_2_FeGe nanoparticles with <10 nm in diameter and revealed that the partial formation of excess Co sites must be excluded in the synthesis processes if the target is a selective reaction.

## Conflict of interest

The authors declare no conflict of interest.

1

## Supporting information

As a service to our authors and readers, this journal provides supporting information supplied by the authors. Such materials are peer reviewed and may be re‐organized for online delivery, but are not copy‐edited or typeset. Technical support issues arising from supporting information (other than missing files) should be addressed to the authors.

Supporting Information

## Data Availability

The data that support the findings of this study are available from the corresponding author upon reasonable request.
