# Supplementary material for: Co2FeGe Heusler Alloy Nanoparticle Catalysts for Propyne Hydrogenation and Ammonia Decomposition
Source: ChemistryOpen. 2023 Nov 6;12(11):e202300131. doi: 10.1002/open.202300131 (PMC10628335; doi:10.1002/open.202300131)
Supplement: Supplementary file 1 — Supporting Information [file OPEN-12-e202300131-s001.pdf]

ChemistryOpen

Supporting Information

Co_2FeGe Heusler Alloy Nanoparticle Catalysts for Propyne Hydrogenation and Ammonia Decomposition

Takayuki Kojima,* Yuki Nakaya, Souta Tate, Satoshi Kameoka, and Shinya Furukawa*

Particle size from TEM image

TEM observation of Co_2FeGe particles synthesized with the Co loading of 1 wt%: sample 2) and 0.5 wt%: sample 3) was challenging, because it was too difficult to distinguish small particles of <10 nm in diameter from nanoporous morphorgy of SiO_2 supports. Thus, the average particle size and the size distribution cannot be estimated from TEM images especially for sample 3). For reference, an attempt to roughly estimate particle sizes of sample 2) is described as below.

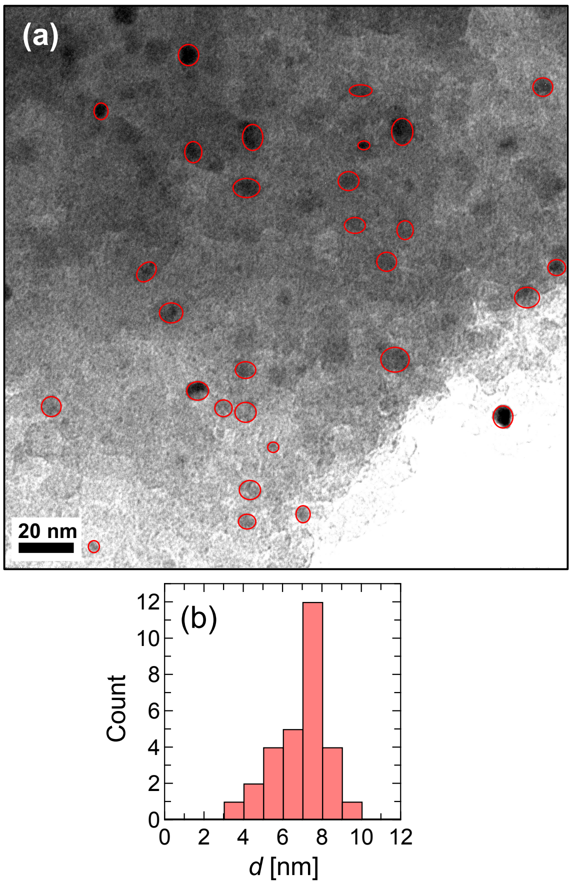

Figure S1. (a) TEM image for sample 2) with ellipses indicating distinguishable particles, (b) histogram of diameter.

Table S1. Area and diameter of each particle in Fig. S1(a).

particle	A [nm^2]	d [nm]
1	45.3	7.6
2	38.6	7.0
3	27.8	5.9
4	24.7	5.6
5	59.9	8.7
6	55.6	8.4
7	11.3	3.8
8	38.0	7.0
9	41.5	7.3
10	53.8	8.3
11	34.8	6.7
12	32.4	6.4
13	39.6	7.1
14	29.9	6.2
15	38.6	7.0
16	53.5	8.3
17	49.1	7.9
18	73.0	9.6
19	36.0	6.8
20	42.3	7.3
21	41.2	7.2
22	30.6	6.2
23	44.3	7.5
24	45.6	7.6
25	12.3	4.0
26	42.6	7.4
27	25.5	5.7
28	27.1	5.9
29	14.2	4.3

As shown in Figure S1(a), particles that can be distinguished from the matrix were traced by ellipses. An area, A of each ellipse was estimated as listed in Table S1 using ImageJ.[1] A diameter, d was calculated by $d = 2(A/\pi)^{1/2}$. The histogram of d is shown in Figure S1(b). The volume-weighted average diameter, d_{ave} was estimated to be 7.5 ± 1.1 nm by $\Sigma d_i^4 / \Sigma d_i^3$, where the error is the unbiased standard deviation. This value is not so different from 9.4 nm listed in Table 1 in the main text. However, considering the number of the distinguishable particles, d_{ave} does not represent the overall average value of sample 2). For sample 3), only a few distinguishable particles were observed, making it impossible to estimate d_{ave}.

[1] W. S. Rasband, ImageJ, U. S. National Institutes of Health, Bethesda, Maryland, USA, https://imagej.nih.gov/ij/, 1997–2023.

XRD measurements after catalytic tests

XRD measurements were conducted after catalytic tests to check structural stability in reaction atmospheres. The XRD patterns for the samples after the C_3H_4 hydrogenation were measured using the incident X-ray of 25 keV as well as the samples before the reaction, while the patterns for the samples after the NH_3 decomposition were measured using Cu-Kα radiation (wavelength, λ = 0.15418 nm). Figure S2 shows the patterns with the scattering vector, $q = (4\pi/\lambda)\sin\theta$. All the peaks of Co_2FeGe have been maintained after the catalytic tests, which indicates that even small nanoparticles of <10 nm in diameter are durable in the reaction conditions. A peak around q = 27 nm^{-1} may originate from an oxide formed in air because of following reasons. It is much sharper than Co_2FeGe peaks. The behavior of this peak is not consistent across all samples that its intensity increased after the C_3H_4 hydrogenation for samples 2) and 3) but decreased for sample 1).

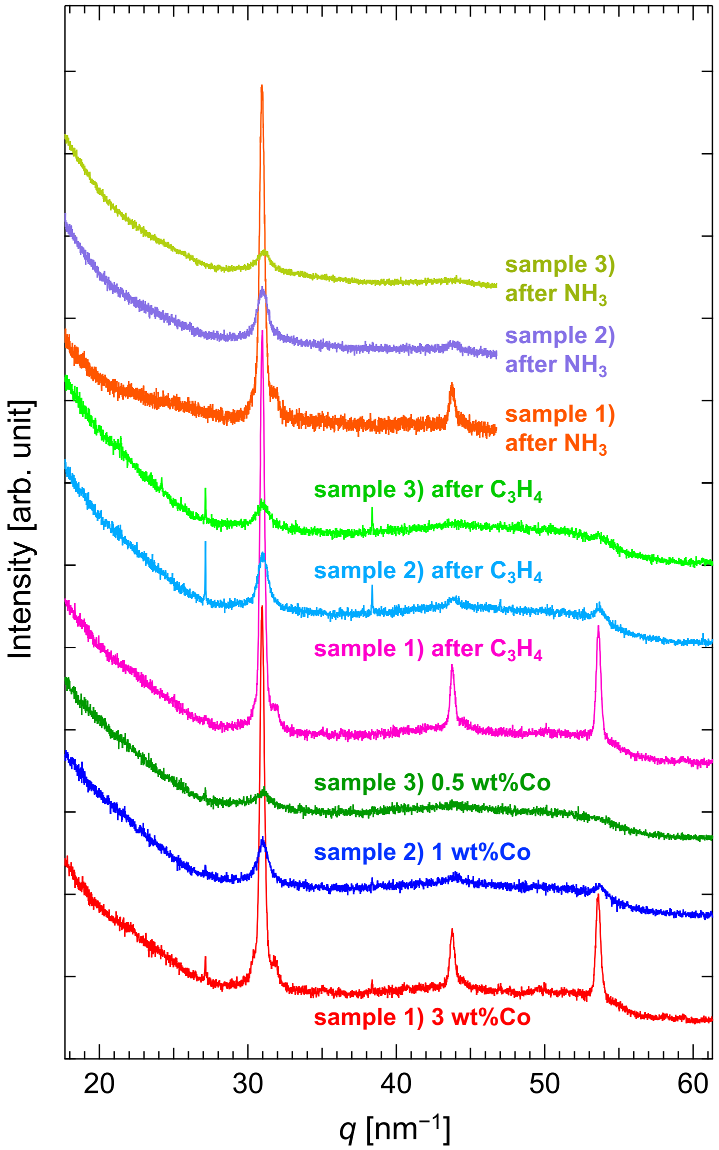

Figure S2. XRD patterns for SiO_2-supported Co_2FeGe nanoparticles, sample 1–3), before and after catalytic tests for C_3H_4 hydrogenation and NH_3 decomposition.

Data of catalytic tests

For data reliability, chromatograms in catalytic tests and analyzed concentrations are shown in Figures. S3 and S4 and Tables S2–S7. In the NH_3 decomposition, quantitative accuracy of H_2 concentration is not guaranteed due to a too weak peak and poor linearity when He is used as a carrier gas.

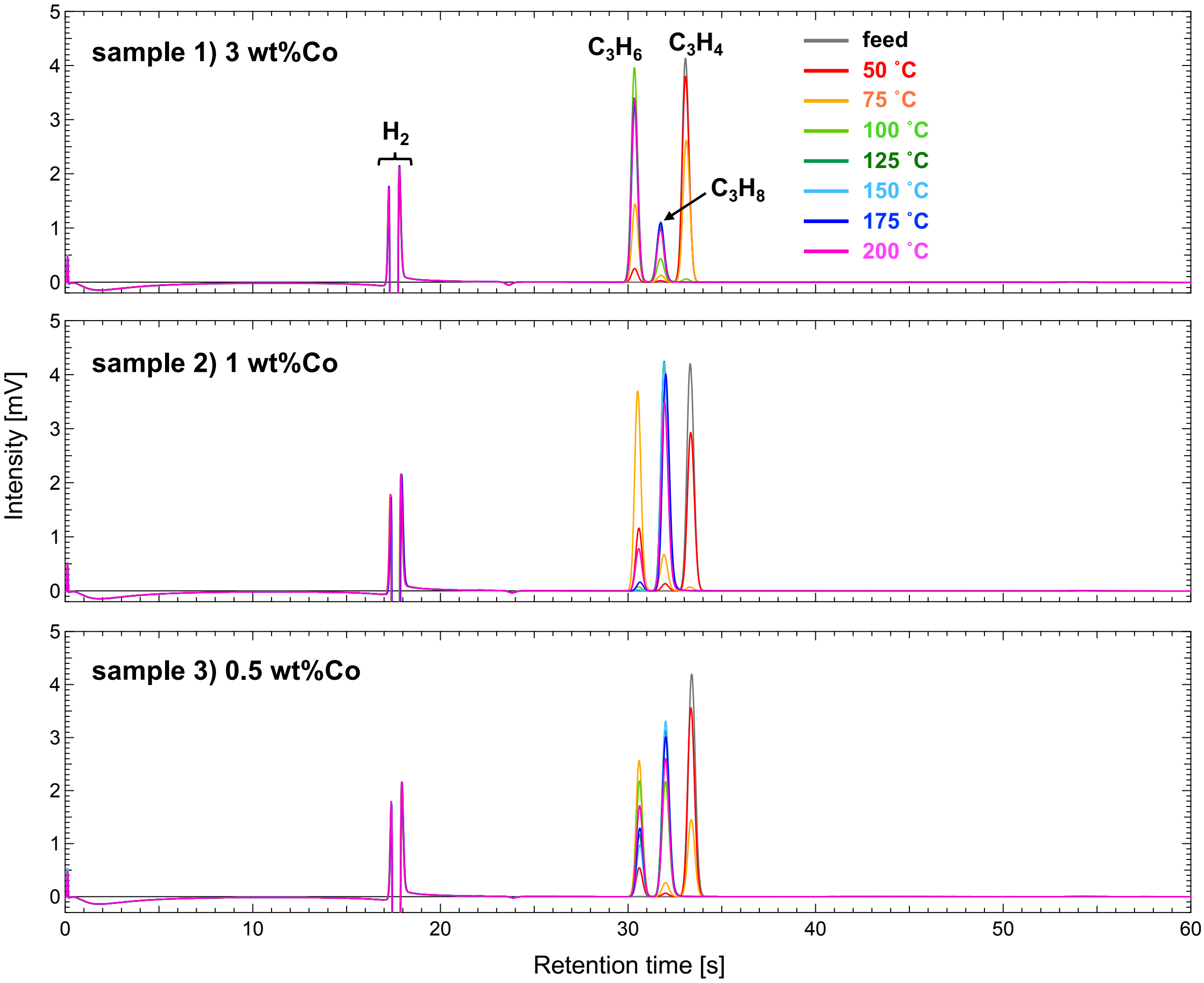

Figure S3. Gas-chromatograms for C_3H_4 hydrogenation by samples 1–3). H_2 makes negative signal with positive signals on both sides. Chromatograms at room temperature are not shown because their raw data were overwritten due to a problem of measurement program (analyzed data have remained).

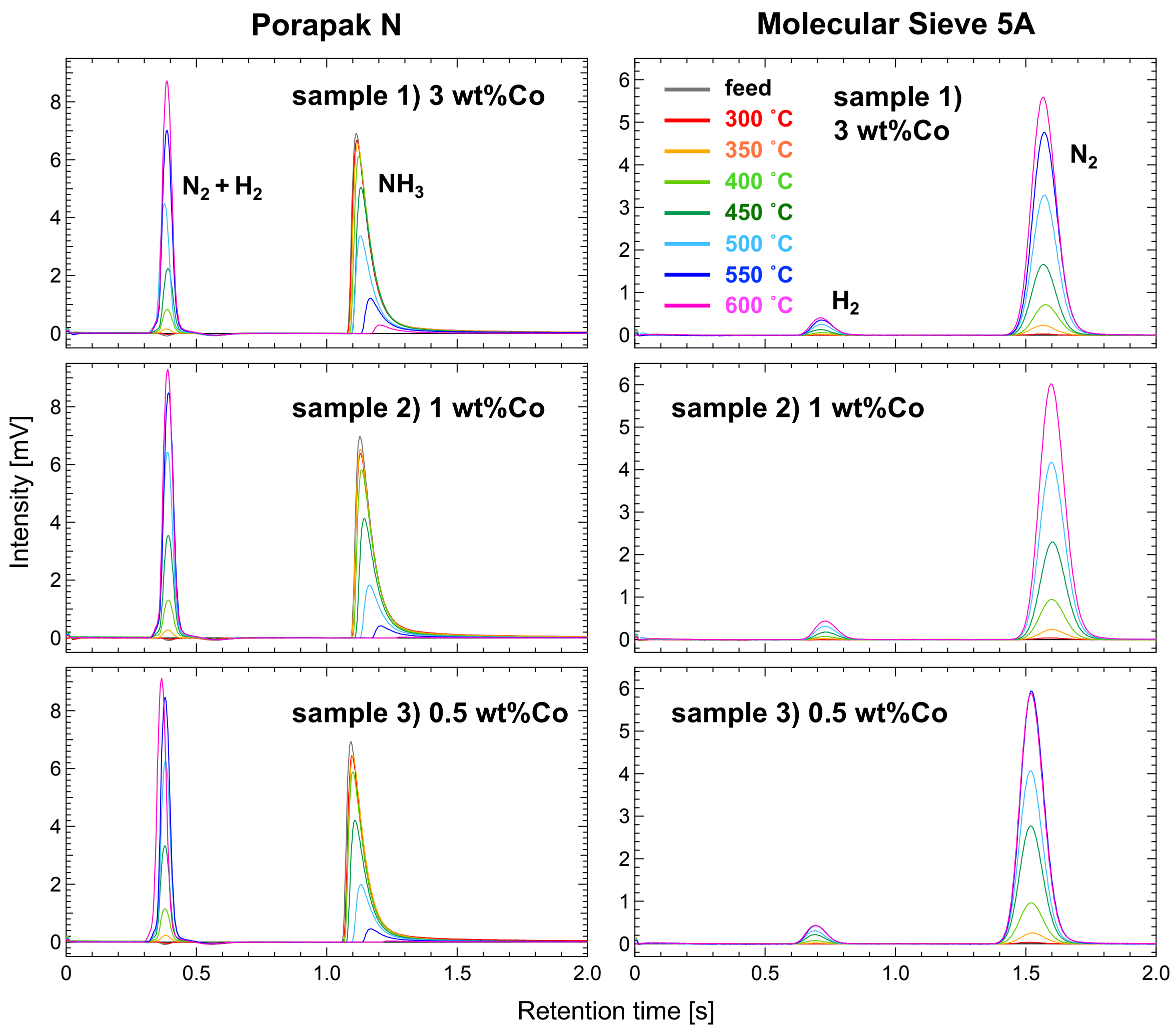

Figure S4. Gas-chromatograms for NH_3 decomposition by samples 1–3). Fluctuation of peak positions is due to manual sampling. Chromatogram at 550 °C using Molecular Sieve 5A for sample 2) is not shown because data acquisition failed.

Table S2. Concentration of hydrocarbons in outlet gas in C_3H_4 hydrogenation by sample 1).

temperature [˚C]	concentration [%]			
	C_3H_4	C_3H_6	C_3H_8	C-lost
24	0.102	0.001	0.000	0.000
49	0.096	0.006	0.001	0.000
75	0.066	0.033	0.003	0.001
100	0.002	0.090	0.011	0.000
125	0.000	0.078	0.025	0.000
150	0.000	0.075	0.027	0.001
175	0.000	0.075	0.027	0.001
200	0.000	0.077	0.024	0.002
feed	0.103			

Table S3. Concentration of hydrocarbons in outlet gas in C_3H_4 hydrogenation by sample 2).

temperature [˚C]	concentration [%]			
	C_3H_4	C_3H_6	C_3H_8	C-lost
22	0.101	0.004	0.001	0.000
50	0.075	0.027	0.003	0.001
75	0.002	0.085	0.016	0.002
100	0.000	0.002	0.102	0.002
125	0.000	0.000	0.103	0.002
150	0.000	0.000	0.103	0.002
175	0.000	0.004	0.098	0.004
200	0.000	0.018	0.085	0.003
feed	0.106			

Table S4. Concentration of hydrocarbons in outlet gas in C_3H_4 hydrogenation by sample 3).

temperature [˚C]	concentration [%]			
	C_3H_4	C_3H_6	C_3H_8	C-lost
24	0.103	0.002	0.000	0.000
50	0.091	0.012	0.002	0.001
75	0.037	0.059	0.007	0.003
100	0.000	0.050	0.053	0.002
125	0.000	0.027	0.076	0.002
150	0.000	0.022	0.081	0.002
175	0.000	0.030	0.074	0.002
200	0.000	0.039	0.064	0.003
feed	0.106			

Table S5. Concentration of gaseous species in outlet gas in NH_3 decomposition by sample 1).

temperature [˚C]	concentration [%]		
	NH_3	N_2	H_2
300	1.03	0.00	0.0
350	1.00	0.02	0.1
400	0.91	0.06	0.2
450	0.78	0.14	0.5
500	0.52	0.28	0.9
550	0.26	0.41	1.3
600	0.08	0.48	1.4
feed	1.03		

Table S6. Concentration of gaseous species in outlet gas in NH_3 decomposition by sample 2).

temperature [˚C]	concentration [%]		
	NH_3	N_2	H_2
300	0.99	0.00	0.0
350	1.01	0.02	0.1
400	0.88	0.08	0.3
450	0.61	0.20	0.6
500	0.31	0.37	1.1
550	0.10	acquisition failed	
600	0.02	0.53	1.6
feed	1.03		

Table S7. Concentration of gaseous species in outlet gas in NH_3 decomposition by sample 3).

temperature [˚C]	concentration [%]		
	NH_3	N_2	H_2
300	1.01	0.00	0.0
350	0.95	0.02	0.1
400	0.85	0.08	0.2
450	0.64	0.23	0.7
500	0.33	0.34	1.0
550	0.11	0.49	1.5
600	0.01	0.49	1.5
feed	1.03		
